# Fat shaming under neoliberalism and COVID-19: Examining the UK’s Tackling Obesity campaign

**DOI:** 10.1111/1467-9566.13555

**Published:** 2022-09-30

**Authors:** Luna Dolezal, Tanisha Spratt

**Affiliations:** 1 Wellcome Centre for Cultures and Environments of Health, University of Exeter, Exeter, UK; 2 School of Humanities and Social Sciences, University of Greenwich, London, UK

**Keywords:** fat shaming, neoliberalism, obesity, public health policy, Tackling Obesity campaign

## Abstract

This article explores the dynamics between fat shaming, neoliberalism, ideological constructions of health and the ‘obesity epidemic’ within the UK, using the UK Government’s recent Tackling Obesity campaign in response to Covid-19 as an illustration. We draw attention to how fat shaming as a practice that encourages open disdain for those living with excess weight operates as a moralising tool to regulate and manage those who are viewed as ‘bad’ citizens. In doing so, we begin by outlining how the ideological underpinnings of ‘health’ have been transformed under neoliberalism. We then consider the problematic use of fat shaming discourses that are often used as tools to promote ‘healthy’ lifestyle choices by those who view it as not only an *acceptable* way of communicating the health risks associated with obesity but also a *productive* way of motivating people with obesity to lose weight. Drawing on Graham Scambler’s theoretical framework regarding shame and blame (2020), we discuss how ‘heaping blame on shame’ has become a ‘wilful political strategy’ under neoliberalism, particularly as it relates to individuals with obesity, and how the Tackling Obesity campaign leverages concerns around ‘choices’ and ‘costs’ as a means through which to encourage normative models of self-care and self-discipline.

## Introduction

In the UK, correlations between excess weight and obesity^1^ with higher rates of hospital admission, serious illness and mortality during the Covid-19 pandemic (PHE report) have led to a renewed ‘fat panic’ (Kirkland, 2010) whereby individuals with excess weight are the recipients of shame, blame and moral outrage for failing not only to maintain their own weight and health, but also for being detrimental to the collective ‘health’ of the National Health Service (NHS). Fuelled by this Covid-19-related ‘fat panic’,^2^ the UK government has launched the Tackling Obesity public health campaign, which aims to ‘empower people to make the healthier choices they want to make’ and lose weight (Tackling Obesity Policy Paper). ^3^ The campaign follows a familiar trajectory of healthism discourses (Crawford, 1980), whereby individuals are seen as personally responsible for their lifestyle ‘choices’ and, hence, their subsequent health status and health outcomes.

Throughout this article, we use the work of Fat Studies scholars alongside medical arguments concerning the health risks associated with obesity to highlight the diversity of these opinions and place them in conversation with each other. In doing so, we show how the Tackling Obesity campaign is exclusively predicated on the medical correlation between excess weight and high-risk status, and consequently fails to meaningfully adopt and/or implement any clear understanding of the socioeconomic and political factors that underpin some of the key concerns that people who are living in larger bodies have. We explore the dynamics between fat shaming, neoliberalism, ideological constructions of health and the ‘obesity epidemic’ within the UK, using the UK Government’s Tackling Obesity campaign as an illustration. ^4^ We draw attention to the ways in which fat shaming, as a practice that encourages open disdain for those living with excess weight, operates as a moralising tool to regulate and manage those who are viewed as ‘bad’ citizens (LeBesco, 2004).

We begin by outlining how the ideological underpinnings of ‘health’ have been transformed under neoliberalism. We then consider the problematic use of fat shaming language that is often used as a tool to promote ‘healthy’ lifestyle choices by those who view it as not only an *acceptable* way of communicating the health risks associated with obesity but also a *productive* way of motivating people with obesity to lose weight (Brown & Baker, 2013; Spratt, 2022). Drawing on Graham Scambler’s theoretical framework regarding shame and blame (2020), we discuss how ‘heaping blame on shame’ has become a ‘wilful political strategy’ under neoliberalism, particularly in relation to individuals with excess weight or obesity. Turning to consider the UK’s Tackling Obesity campaign, we discuss how the campaign utilises stigmatising language and simplistic ideas regarding weight gain/loss that implicitly blames and shames individuals with excess weight for their own poor health outcomes, whilst also explicitly blaming and shaming these individuals for putting strain on NHS resources during a public health crisis. We argue that the Tackling Obesity campaign leverages fat shaming as a means through which to encourage normative models of self-care and self-discipline, explicitly framing obesity as the result of individual ‘choices’, rather than recognising that it is deeply co-implicated with complex societal problems such as poverty, food insecurity, inequality and social deprivation.

## Neoliberalism, Health Policy and Obesity

Neoliberalism is a set of ideological practices that are applied to the economic market and to social life. As David Harvey argues, neoliberalism intrinsically connects the human subject with economics, proposing that ‘human wellbeing can be best advanced by liberating individual entrepreneurial freedoms and skills within an institutional framework characterised by strong private property rights, free markets and free trade’ (Harvey, 2005, p. 2). Under neoliberalism, individuals are defined primarily as consumers who are in competition with others for resources and who are rewarded for hard work and entrepreneurship. In this way, the market is supposed to ensure that each individual receives what he or she deserves because each individual is responsible for their own success and/or failure (Swales et al., 2020, pp. 2–3). As Brown and Baker argue, ‘[w]hilst once neoliberalism might have been about economics, and premised on an ethos of ‘small government’ and liberalised opportunities for entrepreneurs and investors, it has more recently come to embrace desired modes of conduct in enterprising, self-responsible citizens’ (Brown & Baker, 2013, p. 26). By describing the onset and implementation of neoliberal policies in this way, both authors demonstrate how Western countries that have adopted neoliberalism as a primary mode of governance have also adopted new ways of thinking about body image, self-regulation and self-control (Brown & Baker, 2013). This, in part, is because neoliberal ideologies have infiltrated health-care financing and the health-care services of many Western countries through reforms that are focussed on privatisation and weakening national health-care systems (Bambra, 2019, p. 20).

Not only have neoliberal reforms restructured the way that publicly funded health care is organised and delivered, they have also shaped conceptions of what we consider ‘good health’ to be. Under neoliberalism, ‘health’ has become an individual achievement, where the onus of responsibility for good health and health outcomes is largely placed on the individual through an emphasis on the importance of self-reliance and self-control when it comes to lifestyle, behaviour and health outcomes (Brown & Baker, 2013). Following Robert Crawford’s notion of healthism, a form of medicalisation ‘that models popular beliefs, which causes a non-political conception of health promotion by situating the problem of health and disease, and its solutions, at the level of the individual’ (Jiménetcez-Loaisa et al., 2019, pp. 3–4), good health under neoliberalism is a goal that should be achieved through personal investment and commitment and involves an ongoing process that requires constant vigilance and self-restraint. The individualisation of risk and responsibility that comes with neoliberalism means that poor health, along with poverty and other social ills, is often seen as an individual shortcoming and the result of poor lifestyle choices (Schrecker & Bambra, 2015, p. 22). In this way, ‘health’ and ‘health care’ are regarded ‘in much the same way as other consumer goods and services’ (Sturgeon, 2014, p. 414). Individuals are encouraged to find their own solutions to poor health, often through consumer choices within the private sector.

Of the reduction in financial support for public assistance programmes under neoliberalism, the pressure to become self-sufficient and financially independent has become even greater for those who can no longer rely on state support (Schrecker & Bambra, 2015). The term ‘ideal neoliberal citizen’ is currently used to describe a person who is a ‘rational, self-determined agent’ and whose ‘identity is secured by autonomy and choice’ (Shugart, 2016, p. 11). This ‘free self-actualising individual’ (Braedley & Luxton, 2010, p. 11) exercises their autonomy and choice through their consumption of goods that are regulated by a free-market economy. As such, the ideal neoliberal citizen engages in ‘wise’ consumer choices (e.g., healthy foods, gym memberships, etc.) that will maximise their health, and hence their productivity, employability and general success within the market economy. However, one of the tensions at the heart of neoliberalism in everyday life is, of course, that the self-restraint and self-control required to be an ideal neoliberal citizen occurs within an economic and social system, which simultaneously requires individuals to consume more goods than they need (Pirie, 2016). It is a system that, many argue, actively encourages overconsumption and waste (Schrecker & Bambra, 2015). In this way, the subject’s status as an ideal neoliberal citizen is dependent on their ability to self-regulate and demonstrate self-control and self-restraint whilst also spending excessively to support the economy. As a result, as Hannele Harjunen argues, body size and the economy ‘have become closely intertwined with each other’ (Harjunen, 2017, p. 5).

Through being able to exercise self-restraint in terms of food choices and consumption habits, the ideal neoliberal citizen is portrayed as having a particular (‘slim’) body size. Beyond their character traits of self-control and self-determination, they are visually represented as attractive, healthy, affluent, fit and, of course, thin and slender. In contrast, a visible marker of a ‘bad’ or ‘failed’ neoliberal citizen is being overweight or ‘obese,’ where excess weight is seen as an external signifier of one’s presumed laziness, lack of self-control and lack of self-discipline, especially when it comes to food intake and exercise (Fahs, 2017, p. 85). As Amy Farrell notes, fat stigma in the present day is centrally related to ‘anxieties over consumer excess’, where the ‘connotations of fatness and the fat person’ are ‘lazy, gluttonous, greedy, immoral, uncontrolled, stupid, ugly and lacking in will power’ (Farell, 2011, pp. 4, 5). Because of these assumptions, ‘the fat body is constructed as a kind of “anti-neoliberal” body that is unproductive, ineffective and unprofitable’ (Harjunen, 2017, p. 6). In this way, one’s body size has become an immediate and ‘crucial marker of social status’ and a means through which to measure ‘one’s suitability for the privileges and power of full citizenship’ in the dominant economic and social order (Farell, 2011, pp. 2, 5). As Brewis and Wutich note, people with excess weight or obesity find it more difficult to achieve employment, promotion and gain acceptance into university than people who are socially viewed as thin (Brewis & Wutich, 2019a, p. 78). Fat activists and body-positive advocates often highlight these inequities to demonstrate how conversations about weight extend beyond concerns about individual and population-level health and contribute to the widening socioeconomic gap between different people based on their body size.

Whilst some body-positive advocates agree with medical arguments concerning the health risks associated with excess weight and the need for population-level weight reduction, many do not and view the continued health-oriented emphasis on the need for weight loss as both harmful and prescriptive (Hagen, 2019). Moreover, those who hold this view typically see ‘fat’ ^5^ as a bodily marker that is similar to other distinguishing features, including eye colour, hair colour and height. Additionally, some scholars and activists who adopt a similar social justice approach to excess weight point to pre-existing health conditions that are likely to drive weight gain because of the individual’s reduced capacity to regularly exercise and consistently prepare nutritious meals (Toscano, 2019). In this way, excess weight is framed as an outcome of unrelated health conditions rather than as an independent driver of poor health.

Despite the growing popularity of body positivity and fat activism online (Otis, 2020), attitudes suggesting a positive correlation between excess weight and poor health outcomes remain prevalent in contemporary neoliberal contexts (Guthman & DuPuis, 2006; Schrecker & Bambra, 2015). In these contexts, living with excess weight or obesity immediately marks an individual as ‘inferior,’ a citizen who has failed to live up to societal expectations, who is not only failing themselves but also others. These individuals are often blamed for putting strain on public health systems and draining the public economy, unfairly and selfishly taking resources that could benefit others who practise more ‘responsible’ health behaviours (Hopkins, 2012). In the neoliberal order, ‘the fat body has been ranked as an ‘expensive’ body’ (Harjunen, 2017, p. 6). As a result, those carrying excess weight are often viewed as socioeconomic ‘burdens’ who have ‘failed’ to engage in healthy behaviours that will lead to weight loss. As Harjunen notes, ‘The assumed ‘choice’ to be fat (out of moral incompetence) is then used to justify the discrimination and shaming of fat people … the stigmatisation of fatness [is] more widespread, public and socially acceptable. Public monitoring, surveillance and outright ‘policing’ of (fat) bodies by the media, health professionals and even the general public is pervasive’ (Harjunen, 2017, p. 5).

## Conceptualising ‘Fat Shaming’ Within a Neoliberal Framework

Fat shaming is a practice wherein people living with overweight or obesity are purposefully stigmatised and deemed responsible for their body size (Spratt, 2021). Under the logic of fat shaming, these individuals are made to feel *ashamed of,* and *to blame for,* their body size. Graham Scambler identifies ‘attributions of shame and blame’ as central to the successful maintenance of a social order, where the reproduction of the status quo, along with its norms, practices and ideologies, depends on ‘rooting out the misfits in all their heterogeneity and the variety and severity of the threats they represent’ (Scambler, 2020, p. 2). Those who are stigmatised, he argues, ‘infringe against the norms of shame’ and reveal that they have an ‘ontological deficit,’ or in other words, reveal that there is something deficient at the core of their being. In contrast, those who are simply ‘deviant,’ or do not comply with the dominant norms and rules of a society, can infringe the norms of blame; these individuals have a ‘moral deficit’ where they are seen as irresponsible and wilfully non-compliant (Scambler, 2020, p. 84).

In Western neoliberal societies such as the UK, people living with overweight and obesity are often both *shamed* and *blamed* for their body size and any medical issues that might come from it. Using Scambler’s theoretical framework, this ‘heaping blame on shame’ is a ‘wilful political strategy’ where shaming is paired with blaming in order to demonise and stigmatise certain groups. Under the logic of neoliberalism, this renders ‘people personally responsible for their ‘problems’, whatever form these might take’ (Scambler, 2020, p. 79). Individuals who are living with overweight or obesity are believed to have directly caused their body size, along with any related health conditions, through poor lifestyle choices (Garthwaite & Bambra, 2017). This moral deficit, worthy of blame, is presumed to be caused by an individual’s intrinsically flawed character, which signals an ontological deficit that is worthy of shame. ^6^

Contemporary forms of fat shaming are intrinsically bound up with a neoliberal logic, which claims that each individual is responsible for their own self-making and their position in the social order; any failure, misstep or mishap, is shamefully *one’s own fault.* In this way, some theorists argue that shame has fast become the ‘“master emotion” of contemporary neoliberal societies’, where feelings of ‘powerlessness, insecurity, worthlessness, as well as fears of losing one’s status and established living standards’ (Salmela, 2019, p. 186) give rise to a persistent shame anxiety, or fear of being the recipient of blame and shame. As Philip Mirowski notes, the ‘daily spectacle of the public putdown’ (Mirowski, 2013, p. 133) has become a central cultural pedagogy under neoliberalism, evidenced by the predominance of cultural phenomena such as reality television shows, where shaming is utilised as a motivating force to provoke personal transformation towards neoliberal ideals. (It is worth noting that a subgenre of these television shows centres on overweight or obese bodies being shamed into weight loss, for example, *The Biggest Loser, The Big Fat Truth, From Fit to Fat to Fit* and *I Used to Be Fat.)*

The normative contours of contemporary fat shaming and blaming are underpinned by this neoliberal logic, whereby individuals are assumed to be able to *choose* which foods they have access to, and hence can eat or feed their children, and the amount of exercise that they, or their children, can undertake on a daily basis (Brewis & Wutich, 2019a). Moreover, claiming that people living with overweight and obesity are directly responsible for their excess weight is often utilised to support the argument that fat shaming is ‘beneficial’ because it could prompt a change in their ‘poor behaviours’ (Spratt, 2021). As noted by US television host Bill Maher in 2019, ‘some amount of shame is good. We shamed people out of smoking and into wearing seatbelts. We shamed them out of littering and most of them out of racism. Shame is the first step in reform’ (Lee, 2019). This sort of cavalier attitude towards the *explicit* use of shame is often questioned and rejected in public health contexts where it is recognised that shame, blame and stigma can cause personal and social harm, which may worsen health outcomes (e.g., Brewis & Wutich, 2019b). However, the *implicit* use of blaming and shaming, for example, through the use of stigmatising language and overly simplistic ideas about obesity and the antecedents of weight gain and weight loss, is being deployed liberally in contemporary Western contexts. For instance, in 2010, the UK’s Public Health Minister Anne Milton told the BBC that health professionals should use the word ‘fat’ because it (and presumably the shame and stigma it typically provokes) will motivate people to take ‘personal responsibility’ for their lifestyles and motivate them to lose weight (Triggle, 2010).

## The Uk Government’S Tackling Obesity Campaign

A Public Health England Report, released on 25th July 2020, outlined clear evidence that the risk of hospitalisation, intensive care admission and death from Covid-19 was greater for those who were ‘obese’ or severely ‘overweight’ (Public Health England, 2020). The correlation between being overweight and the risk of ending up in an NHS hospital because of Covid-19 was undeniable. Days after the release of this report, the UK government launched a new obesity strategy, published alongside Public Health England’s new ‘Better Health’ campaign, that calls ‘on people to embrace a healthier lifestyle and to lose weight if they need to’ (Tackling Obesity Press Release). The population campaign, targeted at all adults and children, focusses on ‘tackling obesity’ in order to ‘improve the health of the nation,’ ‘offer greater protection against the impact of COVID-19’ and ‘protect the NHS from being overwhelmed’ in the event of a second, or subsequent, wave of the virus (Gasper, 2020, p. 1082). ^7^ The launch of the Tackling Obesity initiative is explicitly linked to former Prime Minister Boris Johnson’s own experience of being overweight when he contracted and became seriously ill with Covid-19 during the Summer of 2020 (Gasper, 2020). To coincide with the launch of this campaign, Boris Johnson spoke candidly about his illness experience in a social media video released to coincide with the launch of the campaign. In this video, Johnson says he was ‘too fat’ and ‘way overweight’ when he was admitted to the hospital. He adds, ‘I’ve always wanted to lose weight for ages and ages … and like … many people, I struggle with my weight’ (Johnson, 2020). In the interview, Johnson describes how, after recovering from his illness, he started jogging in the morning and lost weight as a result of changes to his routine and lifestyle (Johnson, 2020).

The Tackling Obesity strategy targets change on an individual-level, promoting healthy eating, physical activity and weight loss. This approach is not new. In fact, the Tackling Obesity campaign is a further iteration of an ‘anti-obesity’ public health policy in the UK that has been in place for over a decade. The Change4Life ‘anti-obesity’ campaign, which targets childhood obesity in particular, deploys behavioural economics under the guide of ‘nudging’, along with libertarian paternalism, to guide ‘anti-obesity’ public health communication (Mulderrig, 2017, p. 455). This approach retains ‘freedom of choice’ for individuals whilst simultaneously ensuring compliance with desired policy outcomes (Mulderrig, 2017, p. 455). As Mulderrig argues, in relation to the Change4Life campaign, the UK government’s obesity policy discourse interprets obesity as *‘potential future risk* and *economic* threat’ (Mulderrig, 2017, p. 462) and uses this interpretation to legitimise policy decisions. It is precisely this interpretation of obesity that guides the Tackling Obesity campaign within the context of the Covid-19 pandemic, where the potential future risk and economic threat of obesity are channelled directly into concerns about the ‘health’ of the NHS.

The heading of the campaign’s official press release urges individuals to ‘lose weight to beat coronavirus (Covid-19) and protect the NHS’ (Tackling Obesity Press Release). The policy paper outlining the details of Tackling Obesity states that it aims to ‘empower people to make the healthier choices they want to make’ (Tackling Obesity Policy Paper). The injunctions to individual-level change regarding exercise and food choices are coupled with measures that address some societal-level issues, such as ensuring calorie counts are included on some restaurant menus, limiting the advertisement and promotion of unhealthy food in shops and on television and expanding weight management services. However, it should be noted that even these supposedly ‘societal-level’ changes come down to individual choices regarding which foods to consume or purchase, along with which health services to engage with. The campaign is a remarkable change of tack for Boris Johnson, who is well known for his outspoken views regarding the right to unfettered food choices and being opposed to government interventions that promote healthy eating. In 2006, he is reported as commenting on Jamie Oliver’s well-known campaign for healthy school meals by saying, ‘if I was in charge I would get rid of Jamie Oliver and tell people to eat what they like … I say let people eat what they like. Why shouldn’t they push pies through [school] railings … this pressure to bring in healthy food is too much’ (BBC News, 2006).

When Johnson *was* in charge (in stark contrast to his previous position), he sanctioned and supported a national health campaign for which a central goal is to encourage healthier food choices. One advertisement for the campaign shows an older man wearing hi-vis gear—perhaps a construction worker—eating chopped fruit from a plastic tub beside the tag line ‘Healthy eating starts with simple swaps.’ The idea, presumably, is that this individual has simply swapped an unhealthy snack, perhaps a chocolate bar, for this healthier option. Encouraging individuals to make ‘simple swaps’ is a new iteration of the Change4Life ‘smart swap’ campaign, where individuals were encouraged to cut sugar and fat from their diets through simple substitutions (Change4Life Press Release). These sorts of ‘small changes’ that individuals can make are at the heart of the new Tackling Obesity strategy. Boris Johnson is quoted as saying in the press release: ‘Losing weight is hard but with some small changes we can all feel fitter and healthier’ (Tackling Obesity Press Release).

Whilst the goals of encouraging healthy eating and improving general population health are laudable, the Tackling Obesity campaign frames these goals as individually achievable and ignores the structural barriers that prevent some from implementing this advice. Additionally, this campaign highlights some of the problematic conceptions of health and agency that arise from neoliberal rationalities within health discourses. The campaign closely follows a neoliberal conception of health and citizenship, where individuals are positioned as self-actualising with the unfettered capacity to make rational ‘choices’ about their behaviour and lifestyle and, as a result, are personally responsible for their health status and body size. Solving ‘problems’ related to obesity or excess weight is framed as simply ‘a matter of future-oriented individual risk management’ (Mulderrig, 2017, p. 257). Of course, the ‘simple swaps’ that the campaign encourages are scaffolded by a range of socioeconomic contingencies. Most people simply cannot afford to routinely buy the prohibitively expensive tubs of pre-chopped fruit that serve as the visual paradigm for a ‘simple swap’ (Cooke, 2020). Through an emphasis on individual agency, the campaign encourages self-monitoring practices (e.g., calorie counting), which have been shown to ‘exacerbate eating disorder thoughts and behaviours’ and implies that obesity is ‘an individual’s choice or something to be ashamed of’ (BEAT, 2020). In short, the campaign explicitly frames obesity as the result of an individual’s ‘choices’ which in turn ‘cost’ others. Boris Johnson, again quoted in the Press Release, says, ‘If we all do our bit, we can reduce our health risks and protect ourselves against coronavirus - as well as take pressure off the NHS’ (Tackling Obesity Press Release). By framing obesity in this way, the campaign also minimises the agency of those who rationally make the ‘wrong’ choice when it comes to food. In her study of US class differences and unhealthy food consumption, Priya Fielding-Singh notes that poorer parents are more likely to say yes to their children when asked to purchase unhealthy foods than wealthy parents because it offers them an inexpensive way to show their children affection and support. ‘Raising their kids in an affluent environment,’ she argues, ‘wealthy parents were regularly able to meet most of their children’s material needs and wants’ whereas for poorer families ‘[h]onoring requests for junk food allowed [them] to show their children that they loved them, heard them and could meet their needs’ (Fielding-Singh, 2018). In this way, rational choice-making when it comes to purchasing unhealthy foods can signify a response to the material conditions of poverty that positively allows for demonstrations of agency that reinforce and solidify bonds of kinship.

The Tackling Obesity campaign explicitly and repeatedly emphasises *the costs* associated with bodies with excess weight, reinforcing the idea that fat bodies are ‘expensive’, and, as a result, inherently unprofitable and unproductive (Harjunen, 2017, p. 6). The Tackling Obesity government strategy document states that ‘we owe it to the NHS to move towards a healthier weight. Obesity puts pressure on our health service … If all people who are overweight or living with obesity in the population lost just 2.5 kg (one-third of a stone), it could save the NHS £105 million over the next 5 years’ (Tackling Obesity Policy Document). In this way, individuals are expected to regulate their weight not only to benefit their own health, but also to minimise any ‘burden’ or ‘cost’ that they might pose to their local and/or national health-care systems because of it (Brown & Baker, 2013). This sort of discourse positions individuals with excess weight as ‘irresponsible’ and ‘inconsiderate’, not only do their ‘choices’ negatively affect them and their individual health but also the National Health Service because of the additional financial and resource burdens that their excess weight will incur. Furthermore, this discourse of ‘costs’, leads to a general disdain for those with excess weight where many (including the government) lament the use of tax-payer’s money to fund the negative outcomes of ‘personal decisions’ that are perceived as entirely avoidable. Identifying those with a particular body size as putting a financial strain on the NHS and possibly causing harm to others by taking up resources during the pandemic immediately divides people into those who are deserving and those who are not, or those who should be ‘praised’ and those who can be stigmatised, shunned, shamed or ‘mocked’ (Farell, 2011, p. 5).

Discourses concerning the need to ‘protect the NHS’ in the context of Covid-19 not only reinforce the central role that the NHS plays within UK public health but also reframe the issue of the NHS’s financial precarity as one that is caused by ‘irresponsible’ citizens who fail to take responsibility for their health rather than by state disinvestment and the chronic underfunding of NHS services (Maynard, 2017). By framing it as an individual matter, this campaign capitalises on shared understandings of the need for community support during a global health crisis by reinforcing the need for everyone to play their part in the ongoing fight against Covid-19 by practising good health behaviours. Whilst previous campaigns have also stressed the need for individual weight loss to improve national health outcomes, the timing of the Tackling Obesity campaign, coupled with its explicit use of Covid-19 as its key motivating factor, reinforces understandings of shame in people living with obesity who struggle to lose weight by suggesting that they are failing to contribute to the ongoing national effort to fight Covid-19 and are, therefore, putting lives at risk. In other words, because this national effort entreats overweight citizens to practise ‘good citizenship’ by losing weight and lessening the overall burden on the NHS at a critical moment, it suggests that failing to lose weight actively endangers the lives of others who *have* acted responsibly and who require NHS services for reasons that are beyond their control (i.e., the spread of Covid-19). Indeed, the Tackling Obesity government strategy document is explicit on this point: ‘tackling obesity would reduce pressure on doctors and nurses in the NHS and free up their time to treat other sick and vulnerable patients’ (Tackling Obesity Policy Document).

The campaign concretely demonstrates how implicit fat shaming—where ‘heaping blame on shame’ as a ‘wilful political strategy’—is being operationalised within this public health effort. Not only are individuals with excess weight positioned in the discourse as blameworthy for being inconsiderate and irresponsible, literally *costing others* and harming the NHS, they are also positioned as shame-worthy for seemingly lacking the willpower, rationality or social grace to make the right food and exercise choices, ‘simple swaps’ or morning jogs, that will lead to weight loss. Not only is this shaming and blaming strategy surprising in light of the significant evidence in the public health literature showing that a focus on individual choices and using shame and blame strategies, whether implicit or explicit, in obesity campaigns is wholly ineffective (Brewis & Wutich, 2019a), it is also surprising considering the context within which the campaign was launched—immediately after a lengthy national lockdown where most individuals were housebound, with both physical activity and food choices profoundly affected. Significant numbers of people (29% in one study, 48% in another) reported weight gain during lockdown as a result of factors such as increased snacking, increased alcohol consumption, emotional eating to cope with stress and anxiety, difficulty getting to shops to purchase healthy food, less opportunity to exercise and being more sedentary in general (BBC Food, 2020; COVID Symptom Study, 2020; Zeigler et al., 2020). In this way, the idea in the Tackling Obesity campaign that individuals can simply ‘choose’ their food and exercise regimes is immediately undermined by the public health intervention (lockdown), which was rolled out to tackle the very impetus for the campaign (Covid-19). The focus on individual choice and the ‘costs’ of excess weight, especially during the pandemic when many people have struggled with issues around health, stress and finances, sends a message that is largely counterproductive, leading to feelings of failure and shame related to weight stigma. Indeed, evidence suggests that weight-related stigma circulating during the pandemic led many individuals to experience ‘feelings of shame’ where a reluctance to seek help arose from a ‘perception of being “less of a priority than any other condition”‘ (Le Brocq et al., 2020).

Additionally, the roll out of the Tackling Obesity initiative coincided exactly with the Covid-19 related Eat Out to Help Out initiative. Under this government scheme, members of the public were entitled to a 50% discount, up to a value of £10, in restaurants during August 2020, and individuals were actively encouraged to eat out during that month in order to help support businesses and the economy, which had been adversely affected by the pandemic. Many of the restaurants that signed up to take part were fast food chains, such as KFC and McDonalds, which are directly implicated in weight gain and increases in obesity rates (Currie et al., 2010). In this way, the government incentivised citizens to consume more food (often high-calorie and unhealthy food) whilst simultaneously entreating them to consume less and to demonstrate their capacity to be responsible, self-disciplined citizens. As noted by Guthman and DuPuis, this form of neoliberal governmentality ‘produces contradictory impulses such that the neoliberal subject is emotionally compelled to participate in society as both an out-of-control consumer and a self-controlled subject. The perfect subject-citizen is able to achieve both eating and thinness’ (Guthman & DuPuis, 2006). Needless to say, this sort of mixed messaging further undermines public health efforts and leaves individuals with excess weight feeling blameworthy and ashamed.

## Why Fat Shaming Does Not Work

The prevailing cultural belief that fat shaming/blaming can be used as an effective tool to encourage weight loss, which has infiltrated the media, medicine and public health, is problematic in many ways. Firstly, the logic that underpins these shame/blame dynamics is the misapprehension that the conditions necessary to achieve the status of the ‘ideal neoliberal citizen’ are available to all. Of course, the notion that one can just make changes to one’s food and exercise ‘choices’ ignores the structural and socioeconomic contingencies that frame any expression of individual agency. Not all consumers have the same amount of choice when it comes to the food that they can consume and/or the exercise that they can take. Indeed, under neoliberalism ‘individuals make choices under conditions that are not of their own making’ (Braedley & Luxton, 2010, p. 11). Individuals living in poverty or with low incomes, or who have time restraints that mean they do not have the time or resources to prepare healthy meals, often rely on inexpensive ready-made meals that may have low nutritional value. For single parents, this often means that their children will also consume foods that increase the likelihood of them developing obesity because of their relatively poor nutritional value (Hill, 2016). In addition, when consumers have restricted financial budgets, expensive gym memberships are often inaccessible, which means that many have to rely on free and local forms of exercise such as walking and running in order to stay active. For those who live in unsafe neighbourhoods with high crime rates, this is not always possible or advisable, which consequently limits the amount of physical activity that they and their children are able to undertake (Schmidt, 2009).

The constraints on exercise and food choices have been exacerbated during the Covid-19 crisis by lockdowns and the dramatic rise in individuals using food banks due to poverty and job loss. ^8^ Despite these clear structural limitations in the ‘choices’ that individuals can make about food and exercise, especially during Covid-19, there is an implicit discourse of ‘dietary excess and ignorance’ when considering socially vulnerable populations and obesity (Mulderrig, 2019, p. 116). As Mulderrig notes in relation to the Change4Life campaign, ‘“at-risk” subpopulations,’ particularly working class and poor individuals, are singled out through discursive tactics in order to mark out certain food and lifestyle choices as ‘irrational,’ rather than acknowledging the complex structural factors that delimit one’s opportunities and choices, especially in a UK context where a decade of austerity policy has exacerbated and entrenched social inequality (Mulderrig, 2019, p. 116). In this way, following Scambler, blame is paired with shame to demonise a particular group, deflecting attention away from the broader societal factors that are at play in creating poverty, deprivation and other social harms.

Secondly, research shows that, rather than leading to positive behavioural changes, fat shaming often encourages individuals to develop self-destructive behaviours that increase the likelihood of them gaining additional weight (Meulman, 2019). Individuals with excess weight most likely experience heightened levels of chronic body shame (Dolezal, 2015a). Introducing more shame is likely to exacerbate, rather than alleviate, shame and shame-related behaviours (Dolezal, 2015b). It is well theorised that when one feels shame or ashamed, or even when shame is merely anticipated, powerful ‘scripts’, or ‘basic patterns of behaviour that govern our reactions’ to it, are activated (Nathanson, 1992). Many of these ‘scripts’, which help an individual cope with the perceived threats to one’s social bonds and one’s identity that shame experiences provoke, can lead to defensive and self-destructive behaviour patterns, such as withdrawal, aggression, depression, apathy, and even self-harm (Dolezal & Lyons, 2017). For individuals struggling with weight-related shame and stigma, shame often provokes a ‘negative feedback loop’ where shame induces behaviour, physiological responses and social conditions that lead to further weight gain (Brewis & Wutich, 2019a, p. 105). As Brewis and Wutich note, ‘weight stigma actively *undermines* the possibility of weight loss and ultimately leads to longer-term weight gain’ (2019a, p. 105). Additionally, research shows that feelings of shame and stigma can lead to stress responses in the body, which are in themselves weight-inducing (Brewis & Wutich, 2019a, p. 107) and which can also lead to negative health outcomes more broadly (e.g., Pearl et al., 2017).

Using stigmatising language that implicitly shames and blames individuals with excess weight not only for their own poor health outcomes but also for putting strain on national resources, especially during a public health crisis, occludes the structural issues with which excess weight and obesity are deeply co-implicated (Tyler, 2020). Complex societal structures, that create ‘obesogenic environments’ where particular physical, social and economic factors directly contribute to the likelihood that bodies are or will become obese or overweight (Colls & Evans, 2014), such as city planning and school curricula, along with complex social problems, such as poverty, food insecurity, inequality and social deprivation, mean that interventions which primarily rely on a behaviour-change approach will ultimately fail. As Brewis and Wutich note, ‘despite much public education, there is really no good example of any country managing to reverse the obesity epidemic to date … When people are handed the personal responsibility of trying to lose weight, most fail. The current estimates suggest one in 20 people that lose weight manage to keep it off long-term’ (2019a, pp. 100–101). Despite the evidence that the ‘behaviour change model … is a failing strategy’ (Brewis & Wutich, 2019a, p. 101), this is precisely the model that has been taken up in the Tackling Obesity strategy.

Opting for the behaviour-change strategy and focussing on the medicalisation of excess weight means that governments can continue to rely on strategies and solutions that focus on the individual, whether it is pharmaceutical or medical interventions to ‘cure’ individuals, or behaviour-change campaigns, which rely on individual responsibility to make ‘good’ choices to eat less and exercise more. This, as Mulderrig notes, ‘obscures the macro systemic and historical causes of obesity, like the fact that the governance of the global food economy is strongly influenced by regimes of corporate control and profit maximisation which lead to the over-production of cheap, unhealthy foods’ (Mulderrig, 2019, p. 104). Focussing on individuals and their ‘choices’ means there is less pressure to make the necessary structural and societal interventions that the current evidence suggests might actually make a difference (Brewis & Wutich, 2019a). Changes such as redesigning school curricula to include more physical activity, creating walkable cities and making healthy food options more accessible to those who cannot currently afford them offer a clear path to reducing obesity rates and better overall health in the UK, and do not risk shaming individuals for their excess weight. If the UK government is serious about prioritising weight loss in order to combat hospitalisation and death from Covid-19, these are optimal places to start.

## Conclusion

As the previous three decades have attested, neoliberalism has failed to ensure or promote individual or collective health, wellbeing and success, ultimately leading to the ossification of more rigid socioeconomic hierarchies, along with a pervasive politics of personal responsibility, a cultural disdain for vulnerability, dependence and need and increasingly precarious structures in the fabric of social life (Brown & Baker, 2013). Indeed, the previous decades of neoliberal policy have led to austerity, insecurity and inequality, which have undermined public health and well-being (Schrecker & Bambra, 2015). The assumption that individuals are always able to assume personal responsibility for their weight overlooks the myriad ways in which some are prevented from maintaining a ‘healthy’ weight due to social, economic, and environmental factors that are largely beyond their control.

Discursive frameworks that bolster the claim that health can be achieved through individual hard work and determination neglect the myriad ways in which opportunity is a crucial factor in determining the success of any health intervention. If people living with obesity are unable to access healthy foods due to their high cost, or if they do not have the necessary time to prepare healthy meals because of work-related time constraints, then the chances of them benefiting from public health interventions that emphasise ‘choosing’ healthy food options are minimal. Similarly, for those who are unable to afford costly gym memberships, or who live in unsafe neighbourhoods, or those working more than one job to make ends meet, opportunities to simply ‘exercise more’ may be significantly diminished.

Conceptualising the ‘ideal neoliberal citizen’ as one who engages in healthy behaviours gives way to the understanding that those who do not engage in these behaviours are ‘bad citizens,’ which, for many, justifies the shaming of people who are perceived as simply ‘not trying hard enough’ to lose weight. Despite public health evidence that demonstrates that behaviour-change approaches in health campaigns are ineffective, the UK government’s Tackling Obesity campaign emphasises weight loss through individual responsibility, rendering those who remain overweight failures within a neoliberal framework that conceptualises self-help as a choice that all can make to promote better overall health. Unsurprisingly, a recent study conducted by the Social Market Foundation has shown that the Tackling Obesity strategy has been ‘largely ineffective.’ The study stated that ministers placed too much emphasis on ‘individual willpower and not enough on the environmental and economic aspects of obesity’ (Social Market Foundation, 2020). Not only is the Tackling Obesity campaign both unproductive and ineffective, it is irresponsible in light of the available public health evidence on obesity and anti-obesity campaigns (Brewis & Wutich, 2019a). Also irresponsible are official statements from the country’s former Prime Minister, Boris Johnson, which suggest that ‘small changes’ are all that is needed for weight loss. Not only does this claim fail to match public health evidence, it creates a ripe atmosphere for government-sanctioned shame and blame for those who are not able to affect those ‘changes’, or for whom those ‘changes’ do not lead to perceptible weight loss.

## Data Availability

Data sharing is not applicable to this article as no new data was created or analysed in this study.

## Footnotes

1 Terms such as ‘excess weight’ and ‘obesity’ have been widely criticised by self-identified ‘fat activists’ who view medicalised terms as pathologising a non-medical issue, and by patient advocacy groups who view them as inherently shaming and/or stigmatising. In this article, we have used the terms excess weight, obesity, fat and living in larger bodies interchangeably depending on the viewpoint we were examining to reflect the diversity of these perspectives. We fully acknowledge the difficulty in finding a conclusive term that represents and respects all viewpoints. For further information see: Jane Mulderrig (2019) ‘The Language of ‘Nudge’ in Health Policy: Pre-empting Working Class Obesity Through “Biopedagogy”,’ *Critical Policy Studies,* 13(1): 101–121.

2 Note that we use the term ‘fat’ where appropriate to reflect the political use of this word as one that describes a positive marker of difference rather than a pejorative descriptor of a person deemed ‘gluttonous’ and/or ‘lazy.’

3 Note that the term obesity was not problematised by the UK government in the Tackling Obesity campaign despite its contested history.

4 Our analysis of the ‘Tackling Obesity’ policy document and press release is informed by the document analysis methodology in health policy research, in particular, by Bacchi’s ‘what’s the problem represented to be?’ approach. The overall aim in this methodological approach is to identify the implicit ‘solutions’ and ‘deep seated presuppositions underpinning the proposed change’ (Bacchi, 2009, p. x). Our analysis of the Tackling Obesity campaign is undergirded by a problematisation of the ‘problem’ of obesity and the assumptions regarding its consequences, causes and solutions, particularly as related to the NHS and COVID-19. These are considered through a theoretical discussion of neoliberal ideologies and their relation to body size. Our analysis focuses on key concepts and themes of ‘cost’ and ‘choice’ and how these appear in the documents analysed. It should be noted that our contribution is intended to be theoretical, rather than empirical, and our analysis of the ‘Tackling Obesity’ policy document and press release is intended to inform the broader theoretical discussion.

5 The term ‘fat’ has been appropriated by fat activists and some body-positive advocates to denote an indifferent or, in some cases, positive marker of bodily difference. We use the term here to reflect this view.

6 In the case of children who are living with overweight and obesity, parents are often blamed for seemingly failing to regulate their child’s food consumption and exercise habits, thus putting their child’s health at risk; and they are simultaneously shamed for being ‘bad’ parents. Julia Hartley-Brewer writes in the Daily Telegraph: ‘If you are the parent of a fat child, you are a bad parent … if your child is overweight then that is your fault because you are not doing your job as a parent properly. [These parents] are child abusers, in the same way that any parent who deliberately and knowingly harms the health of their child is an abuser’ (Hartley-Brewer, 2015). This sentiment was manifested in an actual ‘anti-obesity’ public health campaign in Australia, which had the tagline ‘Child obesity is child abuse’ (Brewis & Wutich, 2019a, p. 122).

7 Specific policy changes carried out through the Tackling Obesity campaign include banning advertise for high fat, salt or sugar products on TV and online before 9pm, calorie labelling in large restaurants, cafes and takeaways and ending the promotion of high fat, sugar or salt products in stores and online. Additionally, the UK government has announced a commitment to expanding weight management services in primary care, increasing the frequency of behavioural interventions for obesity in primary care and incentivising GPs to refer patients with overweight/obesity to weight management services in every local health-care system.

8 In April 2020, there was an 89% increase in individuals supported by food bank emergency food parcels, along with 107% more children being supported by a food bank compared to the same period the previous year (The Trussel Trust, 2020).
